# New Age Teaching: Beyond Didactics

**DOI:** 10.1100/tsw.2006.368

**Published:** 2006-03-08

**Authors:** Peter D. Vlaovic, Elspeth M. McDougall

**Affiliations:** Astellas Center for Urological Education, University of California, Irvine, CA, USA

**Keywords:** Surgical education, surgical simulators, skills assessment, laparoscopy

## Abstract

Widespread acceptance of laparoscopic urology techniques has posed many challenges to training urology residents and allowing postgraduate urologists to acquire often difficult new surgical skills. Several factors in surgical training programs are limiting the ability to train residents in the operating room, including limited-hours work weeks, increasing demand for operating room productivity, and general public awareness of medical errors. As such, surgical simulation may provide an opportunity to enhance residency experience and training, and optimize post-graduate acquisition of new skills and maintenance of competency. This review article explains and defines the various levels of validity as it pertains to surgical simulators. The most recently and comprehensively validity tested simulators are outlined and summarized. The potential role of surgical simulation in the formative and summative assessment of surgical trainees, as well as, the certification and recertification process of postgraduate surgeons will be delineated. Surgical simulation will be an important adjunct to the traditional methods of surgical skills training and will allow surgeons to maintain their proficiency in the technically challenging aspects of minimally invasive urologic surgery.

## INTRODUCTION

Competency in surgery requires the acquisition of a certain degree of factual knowledge, clinical judgment, interpersonal communication capability, and technical skills. Traditionally, surgical trainees have mastered their techniques by training in the operative theatre. Unfortunately, the potential to learn in the operating room is becoming increasingly limited by factors such as limited-hours work weeks, federal regulations on the degree of attending surgeon participation, and pressure on faculty surgeons to increase productivity. In addition, increased awareness by the general public of medical errors has reduced the tolerance for training surgeons in the operating room. These issues become particularly important in the training of laparoscopic surgery as these procedures are associated with unique challenges, such as a small working space, limited instrument movement, decreased tactile sensation, and counterintuitive manipulation of instruments in a two-dimensional field of view. In an effort to provide preliminary laparoscopic skills and procedures training, animal laboratories have been utilized. However, they are costly, require highly skilled personnel, lack the exact duplication of human anatomy, and offer only a one-time experience. Similarly, cadaveric training is more costly and lacks the effectiveness of training with perfused tissues. As a result of these limitations, interest is increasing for the use of surgical simulation to supplement surgical education and procedure skills training. The potential for surgical simulation to be used in assessing residency applicants, evaluating surgical trainees, and to certifying surgeons is an intriguing hypothesis. This article will review traditional approaches and new concepts in surgical education as they pertain to surgical resident selection, residency training, postgraduate education, and the certification and recertification process.

Before proceeding with a review of surgical education, it is important to define some of the relevant terminology. The Accreditation Council of Graduate Medical Education (ACGME) has mandated that residency training programs must require residents to develop **core competencies** in six areas to the level expected of a new practitioner. The six areas of competency include patient care, medical knowledge, practice-based learning and improvement, interpersonal and communication skills, professionalism, and systems-based practice. **Formative assessment** is done during a program to provide opportunity to improve the quality of student learning and provide evidence of student learning at a particular point in a program. **Summative assessment** is comprehensive in nature and is used to check the level of learning at the end of a program. **Reliability** is a statistical measure of how reproducible training or evaluation data are. **Test-retest** reliability is measured by having the same group of trainees complete the evaluation at two different points in time. **Intraobserver** reliability measures the stability of results from evaluating the same individual by two different methods of evaluation. **Interobserver** reliability is a measure of how well two or more evaluators agree in their assessment of the same individual. **Validity** is determined by how well an evaluation measures what it sets out to measure.

## NEW CONCEPTS IN SURGICAL EDUCATION

### Selection of Surgical Trainees: Assessing Innate Abilities

Learning laparoscopic surgery is associated with unique challenges not encountered in open surgery training. Some investigators have found that operative performance may not improve with practice, suggesting that assessment of innate abilities may be predictive of operative skill[1]. Gettman and colleagues evaluated 20 laparoscopically naïve urology residents with respect to 13 basic performance resources (BPR)[2]. These included reaction time, hand-eye coordination, immediate recall, manual dexterity, grip and shoulder abductor strength, and upper extremity steadiness. These investigators then analyzed the BPR measurements using a nonlinear causal resource analysis (NCRA), developed by them based on a General Systems Performance Theory[3,4]. For each BPR, a minimum score was determined that would enable residents to perform a high-level task successfully (i.e., two laparoscopic porcine nephrectomies), evaluated with a global rating of operative performance scale similar to an OSATS evaluation (objective structured assessment of technical skill)[5]. These investigators found that their NCRA model could predict the expert rating for the surgical procedure performance in 65% of cases. Particularly exact predictions were noted for residents with the lowest scores. Only 10% of the subjects had their surgical performance underestimated by their NCRA model. As a result, these investigators have suggested that BPRs could potentially be used in the selection of residency applicants. In an effort to determine whether postgraduate urologists have an innate predisposition to surgery compared to individuals that do not practice surgery, McDougall and colleagues determined the BPR scores of 57 urology residency applicants to the University of California, Irvine (UCI) and the University of Texas Southwestern, 8 UCI urology residents, 19 urologists with less than 10 years of practice experience, and 61 urologists with greater than 10 years experience. The medical students and residents had significantly higher scores for immediate recall ($p = 0.003$) and grip strength ($p = 0.04$) compared to the practicing urologists. Dominant hand speed was also significantly faster in the medical students and UCI residents compared to the practicing urologists ($p < 0.02$). The upper extremity neuromotor channel capacity, which tests accuracy of hand movements, however, was significantly higher in the practicing urologists compared to the residents and medical students ($p < 0.0005$). In summary, the authors did not find a direct correlation between innate abilities and surgical experience. As a result, they suggested that BPR

measures lack construct validity and are not reliable methods for selecting applicants to surgical programs. The improved accuracy of the postgraduate urologists was felt to be either acquired during training or an age-dependent phenomenon[6].

Psychomotor tests have been shown to be valid selection tools and have prognostic significance in aviation training programs[7,8]. In dentistry, the space relations test (assessing visual spatial ability) and the Crawford Small Parts Dexterity Tester (hand-eye coordination and manual dexterity) have been shown to be strong predictors for students resigning or delaying graduation[9,10]. These findings led Schijven and colleagues[11] to attempt to correlate scores of a battery of aptitude tests with simulator performance in surgical residents. Thirty-three residents with no laparoscopic training took the Abstract Reasoning test (nonverbal reasoning ability and related to IQ), the Space Relations test, the Gibson Spiral Maze test (hand-eye coordination), and the Crawford Small Parts Dexterity tester. After undergoing training on the Xitact simulator (Xitact SA, Morges, Switzerland), they performed the cholecystectomy clip-and-cut module 30 times and had their learning curves generated. Only the abstract reasoning test correlated significantly to simulator performance. These results led the authors to suggest that aspirant laparoscopic surgeons have their abstract reasoning ability tested before deciding to pursue traineeship. In an effort to determine whether surgeons have a higher visual spatial perception than the general population, Risucci[12] compared test scores between 301 surgeons and surgical trainees with those of a normative sample of 251 adults, mainly composed of college students. The following visual spatial tests were analyzed: (1) Localization, the ability to locate points in a two-dimensional space; (2) Orientation, the ability to visualize the rotation and orientation in space of three-dimensional figures when presented on a two-dimensional plane; (3) Form Completion, the ability to identify objects presumably depicted in incomplete silhouette drawings; and (4) Touching Blocks, the ability to envision the depth and structure of three-dimensional objects depicted on a two-dimensional plane. Risucci found that the mean scores of the Orientation and Touching Blocks tests were significantly greater ($p < 0.01$) in the surgeon group compared to the nonsurgical controls. Surgeons were better able than nonsurgeons to envision depth and to manipulate two-dimensional representations of three-dimensional structures mentally. The investigator concluded that such scores be included in career selection discussions with medical students as well as in the competence assessment of surgeons[12].

Another innate characteristic that may be related to laparoscopic aptitude is tonic accommodation, also known as the resting state of the eye, or dark focus. The eyes involuntarily assume an intermediate focus in total darkness or in bad weather. Individuals with a tonic accommodation close to the windshield of their car have greater difficulties with night driving[13]. Shah et al.[14] measured the tonic accommodation in 19 medical students and scored their performance on the “acquire-place” task of the MIST-VR simulator (Mentice AB, Gothenburg, Sweden). In the acquire-place task, the subject grasps an object (sphere) in the virtual environment using a laparoscopic instrument and then places it into a wire frame. Individuals with tonic accommodation values close to the monitor made more errors during the simulator task compared to those with values further away, toward zero. These researchers postulate that students with values close to the monitor may be focusing on the imperfections of the monitor, such as glare, reflections, or scratches, rather than on the operative field; thus limiting their ability to perform laparoscopic surgery effectively.

Finally, Rosser, as cited by Marriott, has observed that surgeons who played video games for at least 3 h/week were 27% faster and made 37% fewer errors than surgeons who did not play video games. Expertise in video gaming as well as in laparoscopic surgery requires a significant degree of hand-eye coordination[15]. Future research may show a correlation between video gaming expertise and surgical competence. If such a correlation exists, video game scores have the potential to be used in the selection of surgical trainees.

In summary, the assessment of innate abilities when considering applicants to surgical programs is interesting and potentially feasible. Although the measurement of basic elements of human performance (BEP) is controversial, psychomotor tests such as abstract reasoning ability and visual spatial ability have been shown to correlate with surgical skill. Video game scores may potentially be used to help program directors select individuals who have the highest likelihood of successfully completing their training. However, further research is needed in this area of surgical education.

## Residency Training: Laparoscopic Skills Training

Surgical training has become more complex with the introduction of endoscopy, laparoscopy, and robot-assisted surgery. The traditional method of evaluating surgical competence has been the In-Training Evaluation Report (ITER). Typically, these are completed by faculty members for each resident at the end of a surgical rotation, and evaluate several core competencies including technical skills, factual knowledge, clinical judgment, and interpersonal communication skills. At the completion of a residency, the ITERs are integrated by the program director to create a final report[16]. However, several problems have been identified with this time-honored approach. First, ITER evaluations have poor inter- and intrarater reliability[17]. Most faculty assessors are not specifically trained as evaluators and commonly commit a “central tendency error” where the evaluator does not utilize the entire scale when evaluating residents. In addition, the assessment of a particular skill or competency is highly influenced by the rater’s overall impression of the trainee, known as the “halo effect”. Finally, since ITER evaluations are usually completed at the end of a surgical rotation, they rely on the evaluator’s recall of the resident’s performance[18]. Although ongoing documentation of a trainee’s performance can decrease this recall bias, the inter-rater unreliability becomes a further limitation over time.

The best technique for measuring and assessing specific surgical skills or techniques is currently considered to be the OSATS evaluation. This is based on the extensively utilized and accepted objective structured clinical examination (OSCE) format and is composed of checklists and a global rating scale. The checklists encompass specific tasks that must be completed during a technique or procedure. A point is given if the task is performed correctly, and no point is given if the task is done incorrectly or not done at all. In addition, a qualitative component of assessment is performed using a global rating scale. The global rating scale consists of a Likert-type scale with scores ranging between 1 and 5. A score of 1 has the poorest performance and a score of 5 has the highest level of performance. For example, in the assessment of a surgeon’s handling of tissue: 1 = the candidate frequently uses unnecessary force or causes damage by inappropriate use of instruments; 2 = the candidate occasionally uses unnecessary force or inappropriate instruments; 3 = the candidate carefully handles tissues, but occasionally causes inadvertent damage; 4 = the candidate handles tissues carefully, but on one occasion caused inadvertent tissue damage; 5 = the candidate consistently handles tissue with minimal damage[19]. Although the OSATS has been shown to have reliability and validity in assessing operative performance, the specific cut-off point that distinguishes the competent from the incompetent surgeon has yet to be defined for specific procedures[18].

The limitation of animal and cadaveric training laboratories has increased the interest in surgical simulation for the teaching of these challenging surgical techniques. Laparoscopic surgery simulators may be classified as model based, computer based, and hybrid. They can be further subdivided into skills oriented or procedure based. Model-based laparoscopic systems usually consist of a box trainer, actual laparoscopic instruments, and a video/camera system. They are relatively inexpensive, use real clinical instruments, and provide similar tactile and image perception as experienced clinically. By repetitively performing a task such as laparoscopic suturing, for example, manual dexterity can be improved. Nevertheless, model-based systems cannot evaluate knowledge nor clinical judgment, and do not attempt to simulate the wide variation in anatomy seen in surgery. Moreover, model-based simulators cannot independently evaluate performance. An experienced laparoscopist must be present during training sessions to ensure that proper technique is learned and reinforced through practice. Computer-based simulators use a virtual reality–created image to mimic the anatomy and surgical environment. Some of the more advanced virtual reality simulators incorporate a degree of haptic, or force feedback, into the simulator to enhance the sensation of a real clinical experience. These simulators may focus on specific skills (e.g., clipping a bleeding vessel) or on an entire surgical procedure (e.g., cholecystectomy).

Computer-based simulators can potentially provide objective measurement of performance without the need for an expert evaluator to be present at the training session. These computer-based simulators can also evaluate cognitive and psychomotor interaction performance and allow the trainees to explore the outcomes of their actions in a totally risk-free environment. As such, errors in the performance of a task or procedure can be identified immediately and the trainee can repeat the task or step without the error. Although these traits are attractive, limitations include high start-up costs and the need for ongoing software upgrades. Hybrid simulators combine the advantages of computers with the benefits of physical models (e.g., use of real-life instruments) to provide a more comprehensive learning experience. However, with all simulators, it is critical that the trainee receive correct initial teaching of the technique or procedure. Otherwise, incorrect techniques will only continue to be performed and errors not appreciated or identified by the naïve trainee. As such, it is critical that an expert direct the trainee through the initial training sessions on the simulator.

In order for simulators to be used as training and evaluation tools, they must have “validity”. In other words, they must teach and measure what they are intending to teach and measure. There are several components to validity testing of surgical simulators. **Face validity** is usually assessed informally by nonexperts and relates to whether the simulator accurately represents what it is suppose to represent. **Content validity** is an assessment of the appropriateness of a simulator as a teaching device as determined by experts and establishes whether the simulator teaches what it is suppose to teach. **Concurrent validity** is the extent to which the simulator’s assessment correlates with that of the gold standard teaching format (i.e., OSATS, described previously) when the two are utilized concurrently**.** **Construct validity** relates to the ability of the simulator performance score to distinguish between novice and expert surgeons. Construct validity is essential before a simulator can be utilized as a testing device. Alternatively, trainees can be followed over time to see if simulator performance increases with clinical experience. **Predictive validity** refers to the ability of the simulator to predict accurately future performance of the individual surgeon in the technique or procedure in the operating room. This is the most arduous of validity testings, but is essential if the simulator is to be used as a formative assessment device.

Several model- and computer-based simulators have been applied to laparoscopic skills training, but few have undergone validity testing on all levels. Of the physical models, the most extensively tested is the MISTELS (McGill Inanimate System for Training and Evaluation of Laparoscopic Skills, Montreal, Canada). The tasks evaluated include peg transfer, pattern cutting, placement of a ligating loop, and intra- and extracorporeal knot tying. Decrossis and colleagues[20] tested 42 subjects on this system (6 general surgery residents from each year of the 5-year program, 6 laparoscopic surgeons, and 6 open surgeons). Significant correlations were observed between simulator scores and resident level of training for peg transfer, pattern cutting, and knot tying. In addition, the mean scores of the laparoscopic surgeons were significantly higher than those of the open surgeons[20]. Finally, a significant improvement in performance scores was observed in 10 residents who were tested 2 years apart[21]. These studies are evidence for construct validity. The MISTELS system has also been shown to possess concurrent validity. Feldman and colleagues[22] demonstrated a correlation between the MISTELS scores of 50 general surgery residents with their ITERs. Fried and colleagues[23] tested 12 third-year general surgery residents on the simulator and on analogous tasks on a porcine model. Residents were subsequently randomized to either 5 weekly practice sessions on the simulator or to a control group (no practice), and were then retested on the simulator and the pig. Scores in the live animal correlated highly with 5/7 of the respective simulator tasks. This finding suggests that the simulator and the porcine model are evaluating similar skills and, therefore, scores on the simulator may be used to predict those on the animal[23]. Finally, the MISTELS system appears to have predictive validity. Fried and collegues[24] assessed 19 surgical residents on their ability to dissect a gallbladder from a liver bed during a cholecystectomy using an OSATS scoring system. The OSATS scores correlated highly ($r = 0.81$; $p < 0.001$) with total scores on the MISTELS system.

With respect to computer-based simulators, the most extensively studied has been the MIST-VR (Minimally Invasive Surgical Trainer - Virtual Reality, Mentice AB, Gothenburg, Sweden). This system consists of 6 tasks requiring various combinations of grasping, translocation, and diathermy. Since this simulator does not attempt to simulate specific laparoscopic techniques such as suturing and knot tying, it has poor face and content validity[25]. Construct validity, however, is supported by the fact that experienced laparoscopists perform tasks significantly faster than novice surgeons, and have superior economy of motion and lower error scores compared to inexperienced laparoscopists[25]. Grantcharov and colleagues[26] compared the MIST-VR skills scores in 14 surgical residents to evaluation scores, in the same group, obtained during a porcine laparoscopic cholecystectomy. They found a significant correlation between the cholecystectomy procedure score as judged by 3 expert evaluators and the MIST-VR task 6 (diathermy) score with regards to the error score and economy of motion; thus supporting concurrent validity of this simulator. Seymour and colleagues[27] were able to demonstrate a correlation between the MIST-VR skills scores and the operating room performance of residents. In this study, 16 surgical residents were randomized to either VR training (diathermy task) or to an untrained, control group. In the simulator trained group, the training was completed when residents achieved a level of performance defined by a group of expert laparoscopists. Both groups then performed a laparoscopic cholecystectomy which was videotaped and subsequently assessed by expert laparoscopic surgeons blinded to the identity of the individual residents. They noted that gallbladder dissection was 29% faster in the VR-trained group. In addition, the control group was 9 times more likely to fail to make progress through the surgical procedure and 5 times more likely to injure the gallbladder compared to the MIST-VR trained group of surgical residents.

Another recently introduced laparoscopic simulator is the LAPMentor$^{TM}$ (Simbionix, Lod, Israel). This simulator consists of 9 basic laparoscopic skills tasks including camera manipulation with 0- and 30-degree lenses (SK1 and SK2, respectively), hand-eye coordination (SK3), clip application with and without the assistance of grasping devices (SK4 and SK5, respectively), bimanual object manipulation (SK6), cutting (SK7), fulguration (SK8), and translocation of objects (SK9). In addition, the LAPMentor$^{TM}$ provides training in the technique of laparoscopic cholecystectomy and has a variety of clinical case scenarios for this procedure. The LAPMentor$^{TM}$ has been shown to possess both face and content validity[28]. At UCI, 103 individuals were tested on all of the 9 basic skills tasks of the LAPMentor$^{TM}$. This included 23 medical students (MS), 24 general surgery and obstetrics/gynecology residents and fellows (R/F), 26 experienced surgeons with less than 30 laparoscopic cases per year (ES < 30), and 30 experienced surgeons with more than 30 laparoscopic cases per year (ES > 30). Experienced surgeons were defined as practicing urologists, general surgeons, or gynecologists performing laparoscopic surgery in their practice. Scoring on the simulator was calculated according to an algorithm predetermined by the manufacturer, and considered both speed and accuracy in compiling a score. The participants in this study had hands-on instruction by a trained individual on each of the basic skills tasks and performed a single practice trial prior to having their skills measured. There was no significant difference in the scores for the 4 study groups for the camera manipulation skills (SK1 and SK2). The ES > 30, ES < 30, and R/F had similar scores in SK3, SK4, SK5, and SK6, but were all significantly better than the scores for the MS ($p < 0.01$). For SK7 and SK9, the ES > 30 and the R/F groups had similar scores that were significantly higher than the scores for the ES < 30 and the MS groups ($p < 0.01$ for SK7, $p < 0.001$ for SK9). For the fulguration task (SK8), the ES > 30 group had significantly higher scores than both the ES < 30 and R/F groups ($p < 0.01$), which had similar scores that were higher than the MS group ($p < 0.001$). These preliminary data suggest that the LAPMentor$^{TM}$ surgical simulator has construct validity. Specifically, skill task 8 can accurately differentiate individuals with various levels of laparoscopic experience[28].

In conclusion, while surgical simulation is increasingly being incorporated into many surgical training programs, the validity testing of these devices remains sparse. It is encouraging that many of these devices are able to provide construct validity by reliably distinguishing between the inexperienced and experienced laparoscopic surgeon. However, continued application of this type of training combined with OSATS evaluation of trainees operative performances in specific surgical procedures will determine if there is a correlation between the simulator performance score and the level of clinical proficiency, or predictive validity.

## Postgraduate Surgical Education

The acquisition of laparoscopic skills requires a longer learning curve than traditional open surgery and has created unique educational challenges for the postgraduate surgeon. In order to facilitate the development of these skills, specific courses on basic and more advanced laparoscopic techniques have been developed. One of the first of such courses was offered by the Department of Urology at the University of Iowa[29]. The course was held over a 2-day period and consisted of 8 h of didactic lecture, two live video case presentations involving a laparoscopic varicocelectomy and a laparoscopic pelvic lymph node dissection, 4.5 h of practice on an animal model, and an additional 4.5 h on laparoscopic simulators. Of the 322 urologists that participated in the course, 166 (51%) responded to a follow-up questionnaire on practice patterns 5 years after course completion. Although 84% of participants performed at least one laparoscopic procedure 1 year after course completion, only 54% were performing laparoscopic cases at 5 years. Only 37% believed that their laparoscopic experience was sufficient to maintain their skills at 5 years compared to 66% at 1 year follow-up. Moreover, 82% of surgeons performed fewer laparoscopic cases than expected. The reasons for this decrease included lack of indications for laparoscopic surgery, increased cost, decreased patient interest, higher complication rates, decreased institutional support, and increased operative time[29].

More recently, at UCI, a 5-day mini-residency program has been offered to postgraduate urologists as one of four modules: (1) ureteroscopy/percutaneous renal access, (2) laparoscopic ablative renal surgery, (3) laparoscopic reconstructive renal surgery, and (4) robot-assisted radical prostatectomy. The program consists of didactic lectures, pelvic trainer and virtual reality simulator practice, animal and cadaver laboratories, and observation of clinical surgeries. Of the initial 32 urologists who underwent the laparoscopic ablative ($n = 17$) and laparoscopic reconstructive ($n = 15$) modules, 100% responded to a follow-up questionnaire on their laparoscopic practice patterns 1–15 months after completing the mini-residency program. Compared to before the course, participants were performing laparoscopic radical nephrectomy ($p = 0.008$), nephroureterectomy ($p < 0.0005$), and pyeloplasty ($p = 0.008$) at significantly higher rates following training. Moreover, fewer of the participants were performing hand-assisted laparoscopy after the course compared to before the program ($p = 0.008$). Partial nephrectomies and adrenelectomies were also performed more often after completing the course, but the differences were not statistically significant. Overall, 81% of the course participants were performing laparoscopic surgery at a mean of 8 months following completion of the mini-residency program. These data suggest that most urologists completing the mini-residency course are able to transfer these skills to the operating room. Further follow-up will determine whether this skill acquisition is durable[30].

Another potential way to improve the ability of postgraduate urologists to acquire laparoscopic skills is for laparoscopic experts to provide proctoring. Rane and colleagues[31] have proctored 6 postgraduate urologists and recommend that each trainee perform 12–15 cases under the supervision of an expert prior to performing the laparoscopic procedure independently. It was recommended that proctors have a minimum laparoscopic experience of 50 cases. Thus far, 5 out of 6 trainees were able to complete the program within 4 months. Although such a mentoring requires a considerable time commitment and places a significant financial burden on the mentor, it appears to be a valuable adjunct in enabling postgraduate urologists to perform laparoscopic surgery successfully[31]. It is of interest that this 4–6 month mentored training program has an equivalent “take-rate” compared to the 5-day, intensive, mini-residency educational program[30].

## FORMATIVE ASSESSMENT AND CERTIFICATION

Several laparoscopic simulators have undergone validation testing on a variety of levels and some are able to distinguish the expert from the novice laparoscopic surgeon[20,25,28]. In addition, some of the laparoscopic simulators have demonstrated a correlation between their performance scores on skills tasks and the level of residency training[20,21]. The MISTELS and the MIST-VR systems can predict resident performance in the operating room compared to their skill test scores[23,24,27]. As such, a potential role of laparoscopic simulators is in the formative assessment of surgical trainees. By developing a national database of simulator performance scores for residents or by creating expected performance levels based on scores determined with expert surgeons, residents at various levels of training and residency program directors would be able to compare their progress during training to a national standard. This would also provide a better and more objective evaluation of residents. In addition, laparoscopic simulators could be used to determine when individual residents have achieved the required competency, based on nationally established standards, to advance to hands-on, clinical participation in the operating room. In this regard, Teber and colleagues[32] determined the mean time required for four expert laparoscopic surgeons to complete five tasks of increasing difficulty, related to laparoscopic radical prostatectomy, on a pelvic trainer model. The tasks included: (1) simple hand-eye coordination (passing a rubber band through pins); (2) cutting, suturing, and knot tying of linear incisions; (3) cutting, suturing, and knot tying of curved incisions; (4) dorsal venous complex stitch simulation; (5) suturing and knot tying of a tubular structure. In addition, the time required to complete a laparoscopic porcine urethrovesical anastomosis was measured. These procedure times for the experienced surgeons were then multiplied by two to establish a defined time goal for the tasks for the residents during training on the same model. Ten surgical residents then had their baseline task completion times measured. They practiced the simplest task on the models until they were able to complete it properly within the established time goal. With satisfactory completion of the task time goal, the residents sequentially moved to the next level of complexity task for training. When all of the tasks were completed within the specified time goals and met defined standards for each task, the program was considered complete. These investigators determined that mean training time of 40 h was necessary for the residents to progress through the entire program such that they could complete an accurate porcine urethrovesical anastomosis in 30 min. The suturing and knot tying times for the residents increased by 72 and 38% from the baseline, respectively, and this was statistically significant ($p < 0.05$). By achieving a defined level of laparoscopic skill on the simulator model, the investigators concluded that the residents would be better prepared to participate in laparoscopic reconstructive cases in the operating room[32].

Another potential role for surgical simulators is in the summative assessment of surgical trainees and the certification of surgeons. As mentioned earlier, simulators have many advantages. Computerized surgical simulation systems may possess the ability to provide truly objective evaluation and assessment of surgical skills, devoid of race, gender, or age bias. Although most simulators are task oriented and test skill performance, some can evaluate full laparoscopic procedures. However, surgical simulation can create clinical scenarios that simultaneously evaluate clinical judgment while testing surgical skills[27]. Before a surgical simulator can be accepted as a certifying tool, it must undergo rigorous validity testing and be determined to be reliable[23,27]. Finally, the level of performance that is considered “competent” remains to be defined[18]. The current gold standard for surgical skill evaluation is the OSATS score. Prior to implementing simulators as certifying tools, the acceptable OSATS score for specific procedures that equates to proficiency needs to be delineated clearly. This proficiency can then be implemented as a recognized standard of skill performance for surgeons at all levels of surgical experience. Currently used simulator variables include time for task completion, economy of motion, complication rate, and error score. It is yet to be determined the weight each of these variables have in the overall proficiency score.

## CONCLUSION

Surgical simulation is increasingly becoming an adjunct to the traditional methods of surgical skills training in the operative room. There are a multitude of both model- and computer-based simulators that have been extensively validated. As virtual reality technology improves, simulators that train entire urologic procedures will become available. They will be able to replicate the large variation in human anatomy seen in surgery as well as focus on measuring clinical judgment rather than dexterity. Simulators will provide an opportunity for individuals to train repetitively in a totally risk-free environment at their own pace. Incorporating surgical simulation into a well-structured and comprehensive surgical curriculum will enhance the expert educator's role in the training process. Simulation will standardize the teaching of basic surgical skills and will allow the expert clinician to concentrate on teaching good clinical judgment and imparting sound ethical principles that constitute a good surgeon.

The awarding of privileges to perform specific procedures in the operating room will no longer be a right determined by the amount of time spent in a training program. Rather, it will be a true privilege, earned by demonstrating proficiency in the performance of that procedure. This will create uniformity in surgical abilities and minimize errors, thereby reducing health care costs to the overall benefit of our patients.

## BIOSKETCHES

**Elspeth M. McDougall, MD**, joined the University of California, Irvine, Department of Urology faculty in 2002 to continue her clinical and research work in minimally invasive urologic surgery and assist in the development of a minimally invasive surgery education center. She is internationally recognized for her laboratory and clinical research in urologic laparoscopic surgery and for teaching courses on fundamental and advanced endourological and laparoscopic techniques.

Dr. McDougall completed her medical training at the University of Calgary and the University of Ottawa in Canada, and then undertook a fellowship in endourology and extracorporeal shock wave lithotripsy (ESWL) with Dr. Clayman at Washington University School of Medicine. She joined the faculty at Washington University Medical School in 1991, where she spent 9 years in academic urology. Subsequently, she developed the Endourology/Laparoscopic Urology Program at Vanderbilt University in Nashville, Tennessee, during her tenure there as Professor of Urologic Surgery. She is a Fellow of the Royal College of Surgeons of Canada (Urology) and certified with the American Board of Urology. Dr. McDougall has published more than 175 peer-reviewed journal articles and numerous book chapters. She is the coeditor of two textbooks on laparoscopic surgery.

Dr. McDougall was chairperson for the Ad Hoc Surgical Simulation Committee and is a member of the Laparoscopic Training Committee of the American Urological Association. She is on the editorial boards of the *Journal of Endourology* and the *Journal of the Society of Laparoendoscopic Surgeons*. She is Past President of the Society of Laparoendoscopic Surgeons and is a member of the Surgical Simulation Committee of this society. She is a member of the World Congress of Endourology's Scientific Meeting Advisory Committee and was recently elected to the prestigious American Association of Genitourinary Surgeons.

Dr. McDougall is Director of the Astellas Center for Urological Education at UCI and developed the ongoing, 5-day, mini-residency training program in minimally invasive urologic surgery for postgraduate urologists.

**Peter D. Vlaovic, MD,** obtained his medical degree and urology residency at the University of Toronto. He is a Fellow of the Royal College of Surgeons of Canada. Dr. Vlaovic is the Minimally Invasive Surgery Education Fellow for the Astellas Center for Urological Education with the Department of Urology at the University of California, Irvine.
